# Diffuse splenic FDG uptake is predictive of clinical outcomes in patients with rectal cancer

**DOI:** 10.1038/s41598-018-35912-4

**Published:** 2019-02-04

**Authors:** Sang Yoon Kim, Chang Mo Moon, Hai-Jeon Yoon, Bom Sahn Kim, Ji Young Lim, Tae Oh Kim, A. Reum Choe, Chung Hyun Tae, Seong-Eun Kim, Hye-Kyung Jung, Ki-Nam Shim, Sung-Ae Jung

**Affiliations:** 1 Department of Internal Medicine, College of Medicine, Ewha Womans University, Seoul, Republic of Korea; 2 Tissue Injury Defense Research Center, Ewha Womans University, Seoul, Republic of Korea; 3 Department of Nuclear Medicine, College of Medicine, Ewha Womans University, Seoul, Republic of Korea

## Abstract

This study aimed to investigate the correlations between diffuse splenic Fluorine-18-fluorodeoxyglucose (^18^F-FDG) uptake on positron emission tomography/computed tomography (PET/CT) and inflammatory markers and to evaluate the prognostic significance of splenic FDG uptake in rectal cancer patients who underwent curative surgery. We retrospectively analyzed the data from 161 patients who underwent splenic FDG PET/CT staging and subsequent curative surgical resection of rectal cancer between July 2006 and September 2014. The spleen-to-liver uptake ratio (S/L ratio) was calculated by dividing the spleen SUV_mean_ by liver SUV_mean_. We found significant positive correlations between the S/L ratio and neutrophil-to-lymphocyte ratio (*P* = 0.013) and platelet-to-lymphocyte ratio (*P* = 0.007). In a Kaplan–Meier analysis, patients with S/L ratio ≤0.815 had a significantly higher recurrence-free survival rate than those with S/L ratio >0.815 (*P* = 0.028). Also, patients with S/L ratio ≤0.731 had a significantly higher overall survival rate than those with S/L ratio >0.731 (*P* = 0.036). In multivariate analysis, higher S/L ratio, as well as male, poor differentiation, higher TNM stage, perineural invasion, and larger tumor size, was independently predictive of cancer recurrence (>0.815 vs ≤0.815, hazard ratio [HR]: 2.04, *P* = 0.046). With regard to OS, S/L ratio was also an independent prognostic factor for death during follow-up (>0.731 vs ≤0.731, HR: 3.81, *P* = 0.017). Our results show significant correlations between S/L ratio on PET/CT and systemic inflammatory markers. Further, S/L ratio was an independent prognostic factor for predicting recurrence and death in patient with rectal cancer after curative surgery.

## Introduction

Fluorine-18-fluorodeoxyglucose (^18^F-FDG) positron emission tomography/computed tomography (PET/CT) has been widely used for staging^1,2^, evaluating treatment response^3^, and predicting prognosis in patients with rectal cancer^4,5^. In the clinical setting, diffusely increased splenic FDG uptake is sometimes observed by chance while interpreting PET/CT. In healthy individuals, FDG uptake in the spleen is commonly decreased as compared with in the liver and dose not change with age^6–8^ or sex^9^. Due to the fact that hepatic FDG is useful as an internal reference in clinical diagnostic settings, increased splenic FDG uptake greater than hepatic uptake is considered an unusual finding^9,10^. Focal splenic uptake mainly represents pathologic findings including primary splenic neoplasm, metastasis, or inflammation/infection such as human immunodeficiency virus infection or infectious mononucleosis^6,7,11^. In contrast, diffusely increased splenic FDG uptake is usually an incidental findings and its clinical significance remains unclear.

The spleen, the body’s largest secondary lymphoid organ, is responsible for mounting the innate and adaptive immune responses to antigens^12^. Inflammation has been known to play a pivotal role in the pathogenesis and progression of rectal cancer^13,14^. Several studies have reported that systemic inflammatory markers including the neutrophil-to-lymphocyte ratio (NLR), the platelet-to-lymphocyte ratio (PLR), C-reactive protein (CRP), and albumin are significantly correlated with poor overall survival (OS) in patients with rectal cancer^15–17^. Additionally, secondary (splenic) erythropoiesis in the red pulp of the spleen can be activated in chronic anemia^18^. Some studies have reported that anemia was associated with diffusely increased splenic FDG uptake in cancer patients on PET scan^19–23^. We hypothesized that the underlying mechanism of diffuse splenic FDG uptake is associated with systemic inflammation. Thus, we aimed to investigate the correlations between diffuse splenic FDG uptake on PET/CT and systemic inflammatory markers and to evaluate prognostic efficacy of diffuse splenic FDG uptake in patients with rectal cancer.

## Materials and Methods

### Patients

**Figure 1:**
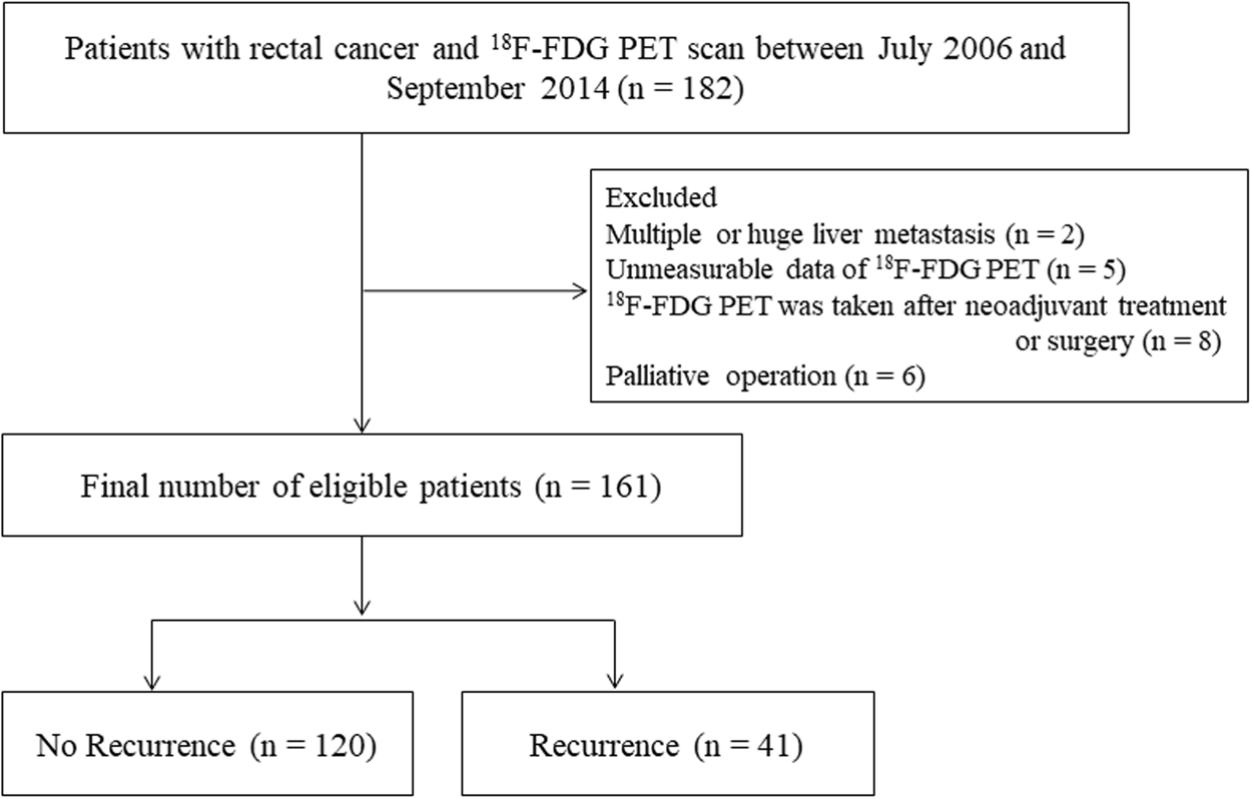
A flow diagram of patient selection in this study. ^18^FDG, Fluorine-18-fluorodeoxyglucose; PET, positron emission tomography.

The electronic medical records of 182 patients with rectal cancer who underwent FDG PET at Ewha University Mokdong Hospital in Seoul, the Republic of Korea were retrospectively reviewed from July 2006 to September 2014. Of these patients, we finally analyzed 161 who underwent PET staging prior to neoadjuvant chemoradiotherapy or curative surgical resection of rectal cancer. None of the patients had concomitant malignancies, such as lymphoma, or acute or chronic inflammatory disease. Patients with synchronous hepatic metastasis were included in the present study if they underwent curative hepatic resection at the same time. Patients were excluded from the current study if they (1) received any neoadjuvant treatment or surgery before PET staging; (2) showed multiple or huge hepatic metastasis, which hamper measurement of the FDG uptake of normal liver parenchyme; (3) underwent palliative surgery; or (4) demonstrated unmeasurable PET data (Fig. 1). The presence of hypertension (HTN) and diabetes mellitus (DM) was determined using the medical records. This study was approved by the Institutional Review Board of Ewha University Mokdong Hospital. Written informed consent was not obtained from participants because this study had a retrospective design. However, patients’ records were anonymized and deidentified prior to analysis to protect their privacy.

### Laboratory studies and cancer staging

Laboratory data, including white blood cell (WBC) (/mm^3^, normal range 4,000–10,000), lymphocyte (%, normal range 20.5–51.1) platelet (/mm^3^, normal range 150,000–450,000), hemoglobin (g/dL, normal range 12.0–16.0), albumin (g/dL, normal range 3.5–5.1), carcinoembryonic antigen (CEA) (U/ml, normal range –5.0), and carbohydrate antigen 19-9 (CA19-9) (U/ml, normal range –27.0) were collected from laboratory studies performed within 10 days before or after ^18^F-FDG PET/CT study. The median period from FDG PET/CT to surgery was 36 days (range 1–237 days). Tumor–node–metastasis (TNM) stages of the study subjects were assessed according to the American Joint Committee on Cancer (AJCC) staging guidelines^24^.

### ^18^F-FDG PET and image analysis

^18^F-FDG PET images were obtained using an Allegro PET scanner (Philips-ADAC Medical Systems, Cleveland, OH, USA) or a Biograph mCT (Siemens Medical Solutions, Erlangen, Germany). Patients were instructed to fast for at least 6 hours before visiting our institutional PET center. About 5.18 MBq/kg of FDG was intravenously injected after confirming the level of fasting blood glucose was less than 140 mg/dL in all patients. Patients were asked to rest for 60 minutes prior to image acquisition. The covering range of PET or PET/CT was from the skull base to the thigh with the patient in the supine position. For PET, a transmission scan using the point source of ^137^Cs was performed for attenuation correction, which was then followed by an emission scan. For PET/CT, a low-dose CT scan was obtained without contrast enhancement for attenuation correction and, subsequently, an emission scan of 2 minutes per bed positions using a three-dimensional mode was acquired and reconstructed using a three-dimensional OSEM iterative algorithm. The PET images of all patients were interpreted by two experienced nuclear medicine physicians who were unaware of the clinical outcomes. Regions of interest (ROIs) confined to the spleen were drawn, and mean standardized uptake values (SUV) were measured and defined as spleen SUV_mean_. The liver SUV_mean_ was measured in ROIs placed on the right lobe of the liver. To prevent respiratory motion-induced artifacts and uptake by adjacent organs (*e*.*g*., the bowel), the ROI was placed in the middle part of the organ in the cranio-caudal direction^21^. In patients with a single hepatic metastasis, the liver SUV_mean_ was measured in a ROI that did not encompass metastatic lesions. The spleen-to-liver uptake ratio (S/L ratio) was calculated by dividing the spleen SUV_mean_ by liver SUV_mean_ for each patient. For the precise measurement of metabolic activity, the placement of ROI was carefully determined and adjusted to avoid possible spillover uptake from neighboring tissue (e.g., the bowel).

### Statistical analysis

Spearman correlation coefficients were calculated for the S/L ratio with regard to NLR, PLR, WBC, hemoglobin, and albumin levels. Differences in variables between patient groups (recurrence vs no recurrence) were analyzed by using the Student’s t-test, chi-squared test or Mann-Whitney U test. Recurrence-free survival (RFS) and OS rates were analyzed using the Kaplan-Meier method and compared via a log-rank test. Survival time was defined as the time between the day of surgical resection and the day of cancer recurrence or death. Patients without recurrence or death were censored at the last follow-up visit. Receiver operating characteristic (ROC) curve analysis of recurrence was performed to assess the optimal cutoff values of continuous variables such as tumor size, NLR, PLR, S/L ratio, hemoglobin, and albumin. Specific cutoff values of all continuous variables in the survival analysis were determined by ROC curve analysis. Aforementioned prognostic factors with *P* values of <0.05 in univariate analysis were included in the multivariate analysis using a Cox proportional hazards regression model which evaluated independent prognostic factors of recurrence or death. The statistical analyses were carried out using MedCalc for Windows version 8.1 and SPSS version 21.0 for Windows software (SPSS Inc, Chicago, IL, USA). Statistical significance was defined as a *P* values of <0.05 under the two-tailed test. Data are available from the corresponding author upon request.

## Results

### Patient characteristics

The characteristics of the 161 patients with rectal cancer in this study are shown in Table 1. Of these individuals, 104 (64.6%) patients were men and 57 (35.4%) patients were women. The mean age of the study participants was 63.6 years ± 11.1 years (range 28–87) in this study. The median follow-up period was 54 months (range 0.4**–**130.3 months). Significant differences were observed in the histopathologic differentiation (poor vs well & moderate, *P* = 0.026), TNM stage (*P* < 0.001), tumor size (*P* = 0.030), lymphatic invasion (*P* < 0.001), perineural invasion (*P* < 0.001), and CEA level (*P* < 0.001) between the patient group with and the group without recurrence. However, the other clinical characteristics including DM, HTN, concomitant therapy and S/L ratio were not different between the two groups.

**Table 1:** Clinical characteristics of the study subjects.

Characteristic	Total (n = 161)	No Recurrence (n = 120)	Recurrence (n = 41)	*p* value^d^
Age, years^a^	63.6 ± 11.1 (28–87)	64.3 ± 10.7	61.3 ± 12.0	0.126
Sex, n (%)				0.995
Men	104 (64.6)	77 (64.2)	27 (65.9)	
Women	57 (35.4)	43 (35.8)	14 (34.1)	
Hypertension, n (%)	41 (25.5)	33 (27.5)	8 (19.5)	0.420
Diabetes mellitus, n (%)	24 (14.9)	19 (15.8)	5 (12.2)	0.756
Histopathology, n (%)				0.026
Well & moderate	153 (95.0)	117 (97.5)	36 (87.8)	
Poor	8 (5.0)	3 (2.5)	5 (12.2)	
T stage, n (%)				0.001
pT1	28 (17.4)	26 (8.1)	2 (4.9)	
pT2	26 (16.1)	24 (2.7)	2 (4.9)	
pT3	100 (62.1)	67 (81.1)	33 (80.4)	
pT4	7 (4.4)	3 (8.1)	4 (9.8)	
N stage, n (%)				<0.001
N0	94 (58.4)	83 (69.2)	11 (26.8)	
N1	36 (22.4)	25 (20.8)	11 (26.8)	
N2	31 (19.2)	12 (10.0)	19 (46.4)	
Distant metastasis^b^, n (%)				0.006
Yes	11 (6.8)	4 (3.3)	7 (17.1)	
No	150 (93.2)	116 (96.7)	34 (82.9)	
TNM stage, n (%)				<0.001
I & II	81 (50.3)	75 (62.5)	6 (14.6)	
III & IV	80 (49.7)	45 (37.5)	35 (85.4)	
Tumor size, cm^a^	4.1 ± 2.5 (0.2–15.0)	3.9 ± 2.6	4.8 ± 2.0	0.030
Lymphatic invasion, n (%)				<0.001
Yes	49 (30.4)	21 (56.8)	25 (21.6)	
No	112 (69.6)	16 (43.2)	91 (78.4)	
Venous invasion, n (%)				0.073
Yes	30 (18.6)	102 (85.0)	29 (70.7)	
No	131 (81.4)	18 (15.0)	12 (29.2)	
Perineural invasion, n (%)				<0.001
Yes	44 (27.3)	97 (80.8)	20 (48.8)	
No	117 (72.7)	23 (19.2)	21 (51.2)	
Concomitant therapy, n (%)				0.084
Surgery only	28 (17.4)	26 (21.7)	2 (4.9)	
Adjuvant chemotherapy	66 (41.0)	46 (38.3)	20 (48.8)	
Adjuvant chemoradiotherapy	33 (20.5)	25 (20.8)	8 (19.5)	
Neoadjuvant chemoradiotherapy	34 (21.1)	23 (19.2)	11 (26.8)	
CEA (U/mL)^c^	3.6 [1.9–8.2]	3.1 [1.8–5.7]	8.7 [3.4–17.6]	<0.001
CA19–9 (U/mL)^c^	9.8 [5.0–20.6]	8.7 [3.4–17.6]	13.0 [5.2–32.5]	0.075
WBC (/mm^3^)^a^	6,854.0 ± 2,283.7 (3,000–13,400)	6792.4 ± 2410.2	7034.2 ± 1879.9	0.560
NLR^b^	2.15 [1.48–2.90]	1.98 [1.39–2.70]	2.33 [1.56–3.58]	0.176
PLR^b^	132.4 [106.3–182.4]	127.8 [106.7–174.8]	147.8 [112.3–194.0]	0.057
S/L ratio^c^	0.783 [0.730–0.828]	0.782 [0.731–0.827]	0.789 [0.736–0.842]	0.233
Hemoglobin (g/dL)^a^	13.0 ± 1.9 (7.1–16.9)	13.0 ± 1.8	13.0 ± 1.8	0.939
Albumin (g/dL)^c^	4.0 [3.6–4.2]	4.0 [3.6–4.2]	3.9 [3.6–4.2]	0.713

Abbreviations: CEA, carcinoembryonic antigen; CA19-9, carbohydrate antigen 19-9; WBC, White Blood Cells; NLR, neutrophil-to-lymphocyte ratio; PLR, platelet-to-lymphocyte ratio; S/L ratio, spleen-to-liver mean standardized uptake ratio; TNM, tumor–node–metastasis

^a^Normally distributed variables are expressed as mean ± standard deviation (range).

^b^Among the 11 patients with distant metastasis, the single liver metastasis was noted in 5 patients, single or multiple lung metastases in 4 patients, and ovary metastasis in 2 patients.

^c^Non-normally distributed variables are expressed as median [25th-75th percentiles].

^d^The t test or Mann-Whitney U test was used as appropriate.

### Correlations between diffuse splenic FDG uptake and inflammatory markers

**Figure 2:**
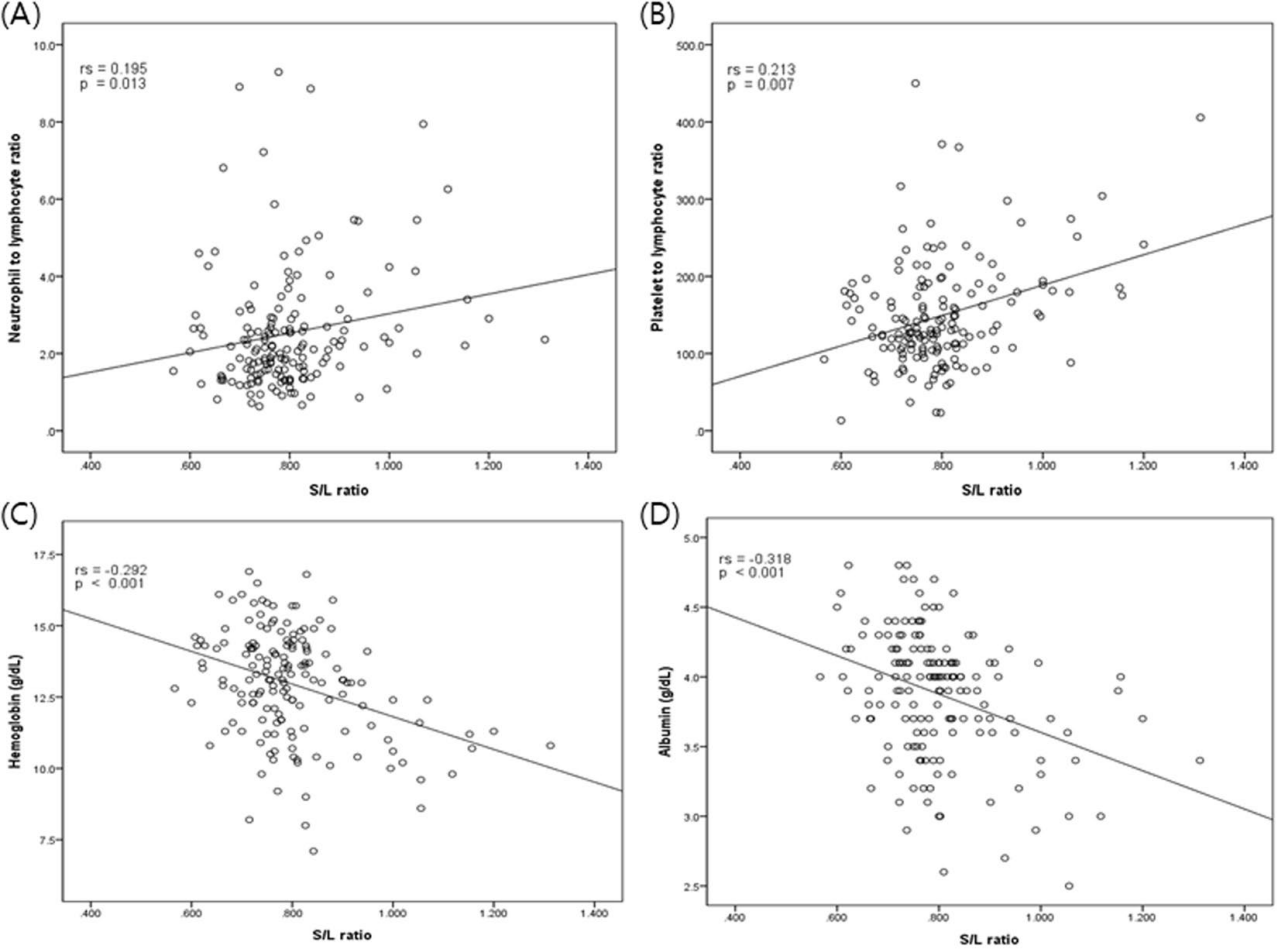
A scatter plot showing the correlations between S/L ratio and hematological and inflammatory markers. S/L ratio, spleen-to-liver mean standardized uptake value; r_s_, Spearman correlation coefficient (**A**) NLR (**B**) PLR (**C**) hemoglobin, and (**D**) albumin.

To evaluate the association between diffuse splenic FDG uptake and systemic inflammatory markers, we assessed S/L ratio, WBC, NLR, and PLR. In addition, we also assessed albumin and hemoglobin (Table 2). We found that S/L ratio was positively correlated with NLR (r_s_ = 0.195, *P* = 0.013, Fig. 2A) and PLR (r_s_ = 0.213, *P* = 0.007, Fig. 2B). In addition, a negative correlation was observed between the S/L ratio and hemoglobin (r_s_ = −0.292, *P* < 0.001, Fig. 2C) and albumin (r_s_ = −0.318, *P* < 0.001, Fig. 2D).

**Table 2:** Correlation between Fluorodeoxyglucose uptake of spleen-to-liver and systemic inflammatory and hematologic markers.

	NLR	PLR	WBC	Hemoglobin	Albumin
S/L ratio	r_s_ = 0.195 *p* = 0.013	r_s_ = 0.213 *p* = 0.007	r_s_ = 0.091 *p* = 0.253	r_s_ = −0.292 *p* < 0.001	r_s_ = −0.318 *p* < 0.001

Abbreviations: r_s_, Spearman correlation coefficients; NLR, neutrophil-to-lymphocyte ratio; PLR, platelet-to-lymphocyte ratio; WBC, White Blood Cells; S/L ratio, spleen-to-liver mean standardized uptake ratio.

### Clinical factors predicting recurrence during follow-up

To evaluate the association of clinical features, systemic inflammatory markers and diffuse splenic FDG uptake with recurrence, Cox proportional hazards regression analyses were performed (Table 3). The optimal cutoff values determined by the ROC curve analysis were 5.0 cm for tumor size, 2.05 for NLR, 177.6 for PLR, 0.815 for S/L ratio, 12.5 g/dL for hemoglobin, and 3.9 g/dL for albumin for the RFS.

**Table 3:** Clinical factors associated with the recurrence during follow-up.

Variable	Univariate analysis			Multivariate analysis		
	HR	95% CI	*p* value	HR	95% CI	*p* value
Age	0.98	0.96–1.01	0.264	0.98	0.95–1.01	0.202
Sex (Male)	1.14	0.60–2.18	0.689	2.33	1.08–5.05	0.032
Histopathology type			0.001			0.026
Well & moderate	1.00			1.00		
Poor	4.74	1.84–12.22		3.59	1.17–11.02	
TNM stage			<0.001			<0.001
I & II	1.00			1.00		
III & IV	7.21	3.03–17.17		6.59	2.48–17.51	
Lymphatic invasion (Yes)	3.42	1.84–6.34	<0.001	1.40	0.60–3.31	0.438
Venous invasion (Yes)	1.94	0.99–3.81	0.054			
Perineural invasion (Yes)	3.67	1.98–6.78	<0.001	2.73	1.17–6.35	0.020
Tumor size (>5.0 cm)	3.51	1.89–6.50	<0.001	5.21	2.61–10.43	<0.001
NLR (>2.05)	2.05	1.06–3.97	0.032	1.16	0.53–2.53	0.716
PLR (>177.6)	2.38	1.28–4.41	0.006	1.57	0.76–3.25	0.226
S/L ratio (>0.815)	1.98	1.06–3.67	0.032	2.04	1.01–4.12	0.046
Hemoglobin (≤12.5 g/dL)	1.14	0.61–2.13	0.686			
Albumin (≤3.9 g/dL)	1.56	0.84–2.90	0.156			

Abbreviations: HR, hazard ratio; CI, confidence interval; TNM, tumor–node–metastasis; NLR, neutrophil-to-lymphocyte ratio; PLR, platelet-to-lymphocyte ratio; S/L ratio, spleen-to-liver mean standardized uptake ratio.

**Figure 3:**
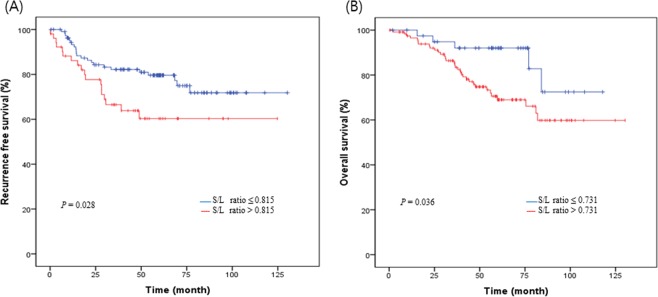
Kaplan-Meier analysis (**A**) Recurrence free survival of patients with rectal cancer in S/L ratio and (**B**) Overall survival of patients with rectal cancer in S/L ratio

In the univariate analysis, histopathologic differentiation (poor vs well & moderate, *P* = 0.001), TNM stage (III & IV vs I & II, *P* < 0 0.001), lymphatic invasion (*P* < 0.001), perineural invasion (*P* < 0.001), tumor size (*P* < 0.001), NLR (*P* = 0.032), PLR (*P* = 0.006), and S/L ratio (*P* = 0.032) were significantly associated with the prognosis of recurrence during follow-up. In particular, on the basis of the Kaplan–Meier survival analysis, patients with S/L ratio ≤0.815 had significantly higher 2-year RFS rates (84.3% vs 77.7%) than did patients with S/L ratio >0.815 (*P* = 0.028, Fig. 3A). Multivariate analysis was subsequently carried out including variables with statistical significance in univariate analysis. Male gender (hazard ratio [HR]: 2.33, 95% confidence interval [CI]: 1.08–5.05, *P* = 0.032), histopathologic differentiation (poor vs well & moderate, HR: 3.59, 95% CI: 1.17–11.02, *P* = 0.026), TNM stage (III & IV vs I & II, HR: 6.59, 95% CI: 2.48–17.51, *P* < 0.001), perineural invasion (HR: 2.73, 95% CI: 1.17–6.35, *P* = 0.020), tumor size (>5.0 cm, HR: 5.21, 95% CI: 2.61–10.43, *P* < 0.001), and S/L ratio (>0.815, HR: 2.04, 95% CI: 1.01–4.12, *P* = 0.046) were independent prognostic factors for recurrence (Table 3).

### Clinical factors predicting mortality during follow-up

The optimal cutoff values determined by the ROC curve analysis were 3.7 cm for tumor size, 2.17 for NLR, 145.4 for PLR, 0.731 for S/L ratio, 11.5 g/dL for hemoglobin, and 3.8 g/dL for albumin for the OS.

In the univariate analysis, histopathologic differentiation (*P* < 0.001), TNM stage (*P* < 0.001), lymphatic invasion (*P* < 0.001), venous invasion (*P* < 0.001), perineural invasion (*P* < 0.001), tumor size (*P* = 0.001), NLR (*P* = 0.010), PLR (*P* = 0.002), S/L ratio (*P* = 0.043), hemoglobin (*P* = 0.011), and albumin (*P* = 0.040) were significantly associated with the prognosis of death during follow-up. Patients with S/L ratio ≤0.731 had significantly higher 2-year OS rates (94.8% vs 92.0%) than did patients with S/L ratio >0.731 (*P* = 0.036; Fig. 3B). The multivariate analysis including significant variables revealed that male gender (HR: 4.22, 95% CI: 1.62–11.00, *P* = 0.003), histopathology (poor vs well & moderate, HR: 5.62, 95% CI: 1.93–16.36, *P* = 0.002), TNM stage (III & IV vs I & II, HR: 4.69, 95% CI: 1.71–12.85, *P* = 0.003), tumor size (>3.7 cm, HR: 2.76, 95% CI: 1.27–5.99, *P* = 0.010), S/L ratio (>0.731, HR: 3.81, 95% CI: 1.27–11.44, *P* = 0.017), and hemoglobin (≤11.5, HR: 2.23, 95% CI: 1.01–4.91, *P* = 0.046) were significant independent prognostic factors for death during follow-up (Table 4).

**Table 4:** Clinical factors associated with the death during follow-up.

Variable	Univariate analysis			Multivariate analysis		
	HR	95% CI	*p* value	HR	95% CI	*p* value
Age	1.03	0.99–1.06	0.109	1.03	0.99–1.06	0.141
Sex (Male)	1.14	0.59–2.21	0.702	4.22	1.62–11.00	0.003
Histopathology type			<0.001			0.002
Well & moderate	1.00			1.00		
Poor	9.40	3.76–23.47		5.62	1.93–16.36	
TNM stage			<0.001			0.003
I & II	1.00			1.00		
III & IV	5.06	2.23–11.50		4.69	1.71–12.85	
Lymphatic invasion (Yes)	5.75	2.90–11.42	<0.001	1.41	0.53–3.80	0.495
Venous invasion (Yes)	3.92	2.05–7.50	<0.001	1.25	0.53–2.94	0.608
Perineural invasion (Yes)	3.70	1.95–7.01	<0.001	2.08	0.91–4.78	0.083
Tumor size (>3.7 cm)	3.56	1.73–7.34	0.001	2.76	1.27–5.99	0.010
NLR (>2.17)	2.48	1.25–4.92	0.010	0.98	0.42–2.28	0.959
PLR (>145.4)	2.82	1.46–5.45	0.002	1.59	0.67–3.78	0.292
S/L ratio (>0.731)	2.64	1.03–6.77	0.043	3.81	1.27–11.44	0.017
Hemoglobin (≤11.5 g/dL)	2.36	1.22–4.57	0.011	2.23	1.01–4.91	0.046
Albumin (≤3.8 g/dL)	1.96	1.03–3.72	0.040	1.29	0.63–2.65	0.487

Abbreviations: HR, hazard ratio; CI, confidence interval; TNM, tumor–node–metastasis; NLR, neutrophil-to-lymphocyte ratio; PLR, platelet-to-lymphocyte ratio; S/L ratio, spleen-to-liver mean standardized uptake ratio.

## Discussion

In the present study, we found a notable association between S/L ratio and systemic inflammatory markers existed. S/L ratio, NLR, and PLR were significantly associated with recurrence and death in the univariate analysis, while only S/L ratio was an independent prognostic factor for recurrence and death in patients with rectal cancer after curative surgical resection. Several studies have reported that the degree of splenic FDG uptake is related to inflammation and hematological parameters^19,21–23,25^. Meanwhile, we considered that the spleen SUV itself would not be appropriate parameter because it can be influenced by various biological and technical factors^26^. Instead, we calculated the S/L ratio by dividing the spleen SUV_mean_ by the liver SUV_mean_, which is widely used as an internal reference. The hepatic FDG uptake is known as an useful internal reference in clinical diagnostic settings due to its homogeneous values^8,9,27^.

In our study, S/L ratio was significantly correlated with NLR, PLR, and hemoglobin as well as albumin. Previous studies have revealed that a significant positive associations of S/L ratio with systemic inflammatory markers such as WBC, neutrophil and CRP were present in patients diagnosed with lung cancer and cholangiocarcinoma^21,28,29^. According prior investigations, presumably, diffusely increased splenic uptake FDG is considered to be a systemic inflammatory response-related phenomenon^28–31^. The spleen consists of red pulp and white pulp, which are separated by the marginal zone containing macrophages and marginal zone B cells^32,33^. The white pulp is involved in adaptive immunity and the marginal zone is involved in both innate and adaptive immunity^12^. An increased splenic FDG uptake can indicate the activation of immune systems in the spleen and can reflect the presence of active systemic inflammation^34–37^. Inflammation has powerful effect on cancer promoters, facilitating genomic instability, and stimulating angiogenesis^14,38,39^. Among inflammatory cells, neutrophils can contribute to cancer promoters and cancer-activated platelets can drive cancer progression and invasion in rectal cancer, whereas lymphocytes can act on cancer-killing responses^40–42^. Albumin is also related to the cancer cell-induced inflammatory reaction^43^. The level of albumin, high NLR, and high PLR have already been reported as systemic inflammatory markers^39,44,45^. Similarly to ^18^F- FDG uptake of the spleen, ^18^F-FDG uptake of bone marrow (BM) in patients with cancer has been reported to reflect the degree of systemic inflammation^19^. In that study, BM SUV was positively associated with systemic inflammatory markers including NLR, PLR, and CRP^19,31,35^. Further, BM was found to be an independent predictor for recurrence in non-small cell lung cancer, lymphoma, and colorectal cancer^10,23,31,46^.

In this study, we also identified the prognostic value of S/L ratio for predicting recurrence and death in rectal cancer. Regarding this issue, ^18^F-FDG uptake of the spleen has been already found to be significantly associated with death in patients with unresectable cholangiocarcinoma^29^. Furthermore, we reported previously that diffuse splenic ^18^F-FDG uptake is significantly related to the RFS and OS of patients with gastric cancer^25^. In the current study, univariate analyses demonstrated that S/L ratio, NLR, and PLR were significantly associated with recurrence and death. Among them, only S/L ratio was independently predictive of recurrence and death in multivariate analysis. This suggests that S/L ratio can be more helpful in the prediction of clinical outcome as compared with other systemic inflammatory markers. Patients with a high S/L ratio showed worse RFS and OS than did those with a low S/L ratio. Cancer recurrence occurred in 22.3% of patients with a high S/L ratio within 2-years after surgery, whereas it developed in 15.7% of those with a low S/L ratio. Similarly, 8.0% of patients with a high S/L ratio died within 2-years of surgical resection, whereas 5.2% of patients with a low S/L ratio died within that period. We thus hypothesize that among patients with rectal cancer, a higher S/L ratio is predictive of poor prognosis after curative surgical resection. Followed by previous study for gastric cancer^25^, this suggests that diffuse splenic FDG uptake, using hepatic FDG uptake as internal reference, has prognostic utility in patients with cancer of the gastrointestinal tract.

In addition, we found a significant negative correlation between the hemoglobin level and the S/L ratio. Therefore, anemia is predictive of death in patients with rectal cancer. Few studies have reported that anemia is associated with diffuse splenic FDG uptake on PET/CT in cancer patients^21,22,28^. We assume that concurrent anemia during PET/CT study may be one of the causes of diffuse splenic FDG uptake. This assumption can be explained by the fact that spleen is a secondary hematopoietic organ^12,32,33^. Thus, when diffuse splenic FDG uptake is observed on clinical PET/CT study, the degree of anemia in patients with rectal cancer who underwent curative surgical resection should be checked for correct interpretation.

The present study has several limitations. First, the number of the study subjects was relatively small, thus weakening the strength of our results. Further larger scaled studies are needed to clarify the implications of diffuse splenic FDG uptake. Second, to homogenize the patient population, we included 161 rectal cancer patients who underwent FDG PET/CT scan for staging and who had hematological data within 10 days before or after PET/CT scan. We excluded patients with colon cancer in order to avoid heterogeneity in the sample population, which might have led to selection bias. Third, diffuse splenic FDG uptake may be influenced by other factors, particularly hemoglobin level. Because anemia stimulates hematopoiesis, hemoglobin level can be associated with FDG uptake of the spleen^12,32^. Fourth, we did not consider pre- or post-operative chemotherapy or radiotherapy, which may affect the prognosis of patients with rectal cancer. Fifth, hepatic micrometastasis, which cannot be detected by PET or PET/CT, might bias the liver SUV_mean_ data, despite exclusion of patients with multiple or huge hepatic metastases. In patients with a single hepatic metastasis, the liver SUV_mean_ was measured in ROIs that did not encompass metastatic lesions. Sixth, the use of two different PET system, a stand-alone PET and PET/CT, is a major limitation. Though the SUV is the most common method in PET studies, using the parameter is not appropriate in our study because technologic factors from different scanners are known to affect SUV measurement. Thus, we used the SUV ratio (S/L ratio) rather than the SUV itself. Last, information on serum CRP results is needed to confirm the correlations between diffuse splenic FDG uptake and the systemic inflammatory markers. The level of CRP, an important acute-phase protein, provides a more accurate estimate of the inflammatory status than other systemic markers of inflammation^16,19,21,35,45^. In our hospital the CRP level is not evaluated during the preoperative work-up of patients with rectal cancer not suspected to have infection or inflammation. The patients in this study did not have infection or inflammation and, thus, did not undergo measurement of the CRP level.

In conclusion, S/L ratio on FDG PET/CT imaging was significantly correlated with systemic inflammatory markers. Moreover, S/L ratio was an independent prognostic factor for the prediction of recurrence or death in rectal cancer during follow-up after curative surgery. Accordingly, we suggest that physicians need to pay particular attention to patients with a high S/L ratio among those with rectal cancer who undergo curative surgical resection.
